# Single-cell sequencing in non-obstructive azoospermia: insights from primary and re-analysis studies

**DOI:** 10.3389/fendo.2025.1539063

**Published:** 2025-03-19

**Authors:** Zesong Jiang, Junwen Zhang, Zhongjian Qiu, Yufei Zhang, Nan Li, Jianmeng Hu, Zhiguo Zhu

**Affiliations:** ^1^ School of Clinical Medicine, Jining Medical University, Jining, Shandong, China; ^2^ Department of Urology, Affiliated Hospital of Jining Medical University, Jining, Shandong, China; ^3^ Medical Research Center, Affiliated Hospital of Jining Medical University, Jining, Shandong, China

**Keywords:** single-cell sequencing, human testis, non-obstructive azoospermia, spermatogenesis, bioinformatics

## Abstract

Non-obstructive azoospermia (NOA) constitutes one of the most severe forms of male infertility. Recent advancements in single-cell sequencing have significantly contributed to understanding the molecular landscape of NOA in human testicular tissues, elucidating the factors that underpin spermatogenic dysfunction. This technology has improved our understanding of the condition at a cellular level. Concurrently, bioinformatics developments have facilitated the re-analysis of publicly available single-cell datasets, offering novel insights into the disorder. Nevertheless, a comprehensive review integrating primary and re-analysis studies of single-cell sequencing in NOA is lacking. This review systematically evaluates 10 primary studies reporting original single-cell sequencing data of human NOA testicular samples and 22 secondary studies that re-analyzed these published data. We explore single-cell sequencing applications in germ cells, Sertoli cells, and Leydig cells, offering a comprehensive overview of molecular insights into spermatogenic dysfunction. Our review highlights novel findings in secondary studies, including the roles of transcriptional regulators, RNA transcription, endocrine disruptors, and microtubular cytoskeleton, thereby bridging primary studies and re-analysis studies. Additionally, we discussed future research directions and the challenges of translating single-cell research findings into clinical applications. In summary, single-cell sequencing offers a high-resolution, single-cell perspective of NOA testicular tissue, paving the way for innovative therapeutic strategies in male infertility.

## 1 Introduction

Non-obstructive azoospermia (NOA) represents one of the most severe forms of male infertility, with a multifactorial etiology that includes factors such as chromosomal abnormalities (e.g., 47XXY), Y-chromosome microdeletions, infections, medications, trauma, and endocrine disorders (1). However, the majority of NOA cases (approximately 80%) remain idiopathic, collectively known as idiopathic NOA (iNOA) (2). Other common pathological types of NOA include Klinefelter syndrome (KS), Sertoli cell-only syndrome (SCOS), and the Y chromosome AZF region microdeletion (AZF_Del). Currently, pharmacological treatments for NOA are ineffective, and the only available option for patients to achieve biological parenthood is microdissection testicular sperm extraction (micro-TESE) combined with intracytoplasmic sperm injection (ICSI) (3). Despite the expertise available at specialized fertility centers, only 30% of patients with NOA are able to successfully retrieve sperm for assisted reproduction, while the majority of patients rely on sperm banks or adoption (4). The highly heterogeneous nature of testicular cells, comprising both germ and somatic cell types, along with the limited understanding of their development and interactions, remains a significant barrier to developing effective treatments and tools for NOA.

While bulk RNA sequencing has facilitated significant advances in understanding the molecular basis of NOA, identifying genes critical for spermatogenesis and testicular immune homeostasis (5), it remains limited by its inability to differentiate between distinct cell types and states, due to its averaging of gene expression signals from heterogeneous cell populations (6). Furthermore, bulk RNA-seq fails to capture the complexity of intercellular communication, which limits its potential to elucidate the pathophysiology of NOA. In contrast, single-cell sequencing offers a distinct advantage in mapping cellular heterogeneity with high resolution and high throughput, enabling detailed insights into gene expression, cell type identification, genetic alterations, intercellular interactions, and cellular functions within complex tissues (5). In 2018, Wang et al. (7) introduced the first high-precision single-cell transcriptome map of human spermatogenesis, categorizing germ cells into three types of spermatogonia, seven types of spermatocytes, and four types of spermatids. Eight days later, Guo et al. (8) published their transcriptional cell atlas of the adult human testis acquired via single-cell RNA sequencing (scRNA-seq). In this study, they identified five discrete transcriptional/developmental spermatogonial states, including a novel early spermatogonial stem cells (SSCs) state, termed State 0. These two pioneering studies demonstrate the significant potential of scRNA-seq in advancing male infertility research. Since then, numerous single-cell sequencing (especially scRNA-seq) studies focused on NOA have been conducted, leading to significant progress in understanding the condition. With advances in bioinformatics, these datasets have since been re-analyzed, yielding further novel discoveries. Previous reviews have addressed single-cell sequencing applications in human testes (9–11), but these studies often do not specifically focus on azoospermia or the application of single-cell sequencing to NOA. For instance, some reviews have concentrated on testes, germ cells, or SSCs, while others have explored techniques other than single-cell sequencing. Additionally, there is a lack of comprehensive studies that systematically analyze the connection between primary research and subsequent re-analysis study. This review provides a comprehensive summary of new findings from single-cell analyses of testicular tissues from NOA patients and explores the relationship between primary studies and re-analysis efforts. This review aims to offer a detailed and holistic overview of single-cell sequencing applications in NOA, providing researchers in the field with a valuable resource for understanding the single-cell resolution of NOA testes.

## 2 Overview of infertility

Clinical infertility is defined as the failure of a couple to achieve conception after at least 12 months of regular, unprotected sexual intercourse. According to reported statistics, approximately 10-20% of couples are infertile, with 30-50% attribute to male factors (12). Infertility or reduced fertility may be caused by various factors, including testicular dysfunction, genetic abnormalities, endocrinopathies, autoimmune diseases, lifestyle factors (such as obesity), heat exposure, exposure to gonadotoxic agents, and aging.

Approximately 5%-10% of men evaluated for infertility are azoospermic (13). Azoospermia refers to the complete absence of sperm in both the ejaculated semen and the centrifuged sediment, and it is one of the most severe conditions of male infertility. Based on the presence or absence of ductal obstruction, azoospermia can be classified into obstructive azoospermia (OA), comprising about 40% of the cases, and NOA, which constitutes the other 60% (13, 14). The majority of patients with OA can be effectively treated through surgical means. For reproductive tract obstruction, microsurgical reconstruction is the preferred approach. In cases of intra-testicular obstruction or when surgical reconstruction is not feasible, surgical sperm retrieval combined with ICSI or sperm cryopreservation is advised. The majority of patients with NOA have primary testicular failure. The detection of genetic abnormalities in men with NOA is crucial. For men with NOA associated with hypogonadotropic hypogonadism, endocrine therapy is an effective first-line therapy. Micro-TESE is the preferred surgical sperm retrieval method for patients with NOA. For men with varicocele-associated NOA, varicocelectomy should be considered prior to sperm retrieval.

## 3 Literature search

**Figure 1:**
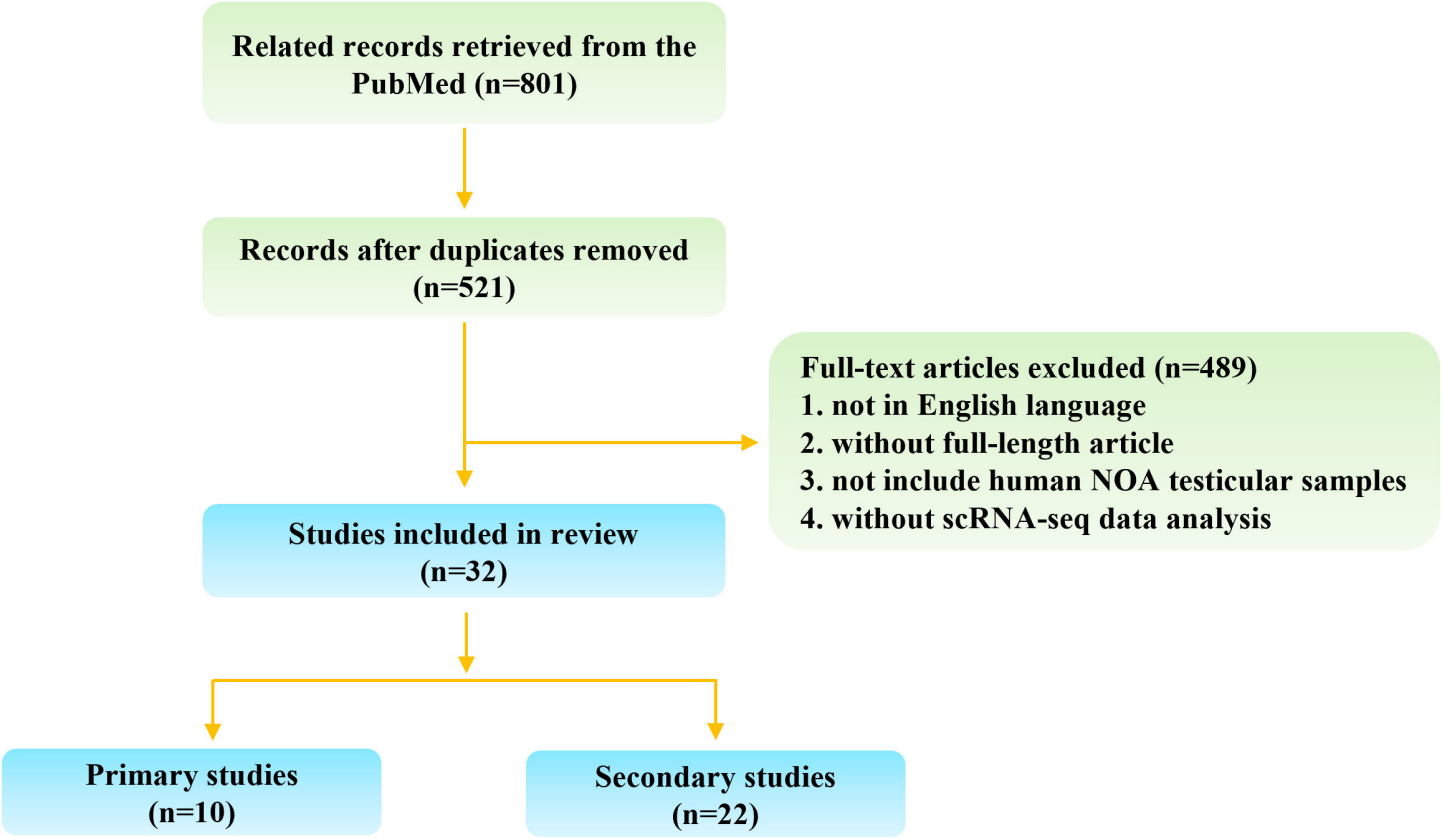
Flow chart of study selection.

A systematic literature search was conducted using PubMed, with no restrictions on publication dates up to August 2024, and limited to studies published in English. To ensure comprehensive coverage, three sets of search terms were used: (“single-cell” [All Fields] OR “scRNA” [All Fields]) AND “spermatogenesis” [All Fields]; (“single-cell” [All Fields] OR “scRNA” [All Fields]) AND “azoospermia” [All Fields]; and (“single-cell” [All Fields] OR “scRNA” [All Fields]) AND “testis” [All Fields]. The inclusion criteria were as follows: (1) single-cell studies using human NOA testicular biological samples, including testicular tissues and cells; (2) studies re-analyzing published or public single-cell sequencing data of human NOA testicular biological samples. The exclusion criteria included: (1) articles not published in English; (2) articles not available in full text; (3) studies without single-cell sequencing data analysis or those not related to NOA. **Figure 1** illustrates the search flowchart outlining the screening process for relevant studies.

## 4 Application of single-cell sequencing in NOA

Single-cell sequencing has emerged as a transformative technology for elucidating the diversity and complexity of RNA transcripts within individual cells, thereby enhancing our understanding of the cellular composition and functional roles of various cell types in tissues (15). Introduced by Professor Tang in 2009, scRNA-seq was first applied to study early human embryonic development (16). In 2018, Wang et al. (7) pioneered the high-precision single-cell transcriptome mapping of human spermatogenesis and characterized the testicular cell types and gene expression profiles in patients with NOA. Leveraging single-cell sequencing technology, Guo et al. (17) identified for the first time in 2020 that prepubertal immature Sertoli cells could be divided into two distinct subpopulations with different physiological states. In the same year, Zhao et al. (18) proposed that Sertoli cells undergo three independent and consecutive developmental stages and, for the first time at the single-cell level, demonstrated that the changes in the testicular somatic cell microenvironment in patients with iNOA primarily involve the maturation blockade of Sertoli cells. Single-cell sequencing has thus ushered in a new era in male infertility research, providing a powerful tool to investigate alterations in the testicular microenvironment, the interactions between germ cells and somatic cells, and the pathogenesis of NOA.

The basic steps involved in single-cell sequencing technology include single-cell isolation, lysis, reverse transcription, cDNA amplification, library preparation, sequencing, and subsequent computational analysis (19). Reverse transcription and amplification of barcoded single cell derived cDNA are critical steps for enhancing the sensitivity and accuracy of single-cell sequencing (20). Prior to analysis, data must undergo quality control, de-batching, normalization, and scaling (21). Quality control is essential due to the higher technical noise present in single-cell sequencing data compared to that of bulk sequencing data. Normalization and scaling are employed to mitigate the effects of differences in sequencing depth (22). Following this, dimensionality reduction techniques are applied. Common methods include Principal Component Analysis (PCA), T-Distributed Stochastic Neighbor Embedding (T-SNE), and Uniform Manifold Approximation and Projection (UMAP) (23). Commonly used analytical techniques include cluster analysis, which is used to identify distinct cell types or subpopulations, while differential expression analysis helps identify genes that are significantly expressed in specific cell types or states. Additionally, pseudotime analysis allows for the reconstruction of cell developmental trajectories or lineage relationships (24).

Through our systematic literature search, 10 primary studies, containing originally reported single-cell sequencing data of human NOA testicular samples, were identified (**Table 1**). Notably, up to 8 out of the 10 primary studies also utilized previously published single-cell sequencing data from normal, OA, or NOA testicular biological samples. These external datasets were employed as normal controls, to increase the sample size of the research, or for external validation purposes. Among the primary studies, the majority (8/10) were conducted by Chinese researchers, with one study from the United States and one from Italy. The distribution of the 10 primary studies (2018-2024) did not exhibit a centralized trend. In terms of sample size, the study by Chen et al. (25) included the largest sample size, with scRNA-seq performed on 17 patients with NOA. Zhao et al.’s study included the most diverse types of NOA, encompassing iNOA, KS, and Y chromosome microdeletions, with a total of 7 patients (18).

**Table 1:** Studies containing originally reported scRNA-seq data of human NOA testicular samples.

Study	Year	Country	Techniques	Samples	External data	Data availability
Wang et al. (7)	2018	China	Modified Smart-seq2	Normal (n=2) OA (n=7) NOA (n=1)	–	GEO: GSE106487
Liu et al. (48)	2020	China	Modified Smart-seq2	NOA (n=1)	GEO: GSE106487	GSA: HRA000146
Zhao et al. (18)	2020	China	10×Genomics BD Rhapsody	Normal (n=10) KS (n=3) AZFa_Del (n=1) iNOA (n=3)	–	GEO: GSE149512
Alfano et al. (31)	2021	Italy	10× Genomics	OA (n=1) iNOA (n=3)	GEO: GSE120508 GEO: GSE124263	GEO: GSE154535
Mahyari et al. (29)	2021	USA	10× Genomics	Normal (n=3) iNOA (n=1) KS (n=2) Secondary infertility (n=1)	GEO: GSE109037 GEO: GSE120506 GEO: GSE130151 GEO: GSE149512	GEO: GSE169062
Wang et al. (30)	2021	China	STRT-seq	NOA (n=1)	GEO: GSE106487 GEO: GSE120508 E-MTAB-6946	GEO: GSE157421
Chen et al. (47)	2022	China	Singleron GEXSCOPE	OA (n=1) NOA (n=1)	GEO: GSE106487 GEO: GSE124263 GEO: GSE112013	NODE: OEP000778
Chen et al. (25)	2023	China	Modified STRT‐seq	iNOA (n=17)	GEO: GSE106487 GEO: GSE149512	GSA: HRA001477
Huang et al. (27)	2023	China	STRT-seq scCOOL-seq	single-cell multi-omics sequencing: Normal (n=4); NOA (n=2). scRNA-seq: NOA (n=9)	GEO: GSE157421 GEO: GSE106487 GEO: GSE109037	GSA: HRA000148 GSA: HRA004917 GEO: GSE235324
Wu et al. (28)	2024	China	Singleron GEXSCOPE	OA(n=6) NOA (n=6)	–	–

In these studies, researchers employed a variety of single-cell sequencing technologies, which include STRT-seq, Smart-seq2, Modified Smart-seq2, Modified STRT-seq, Singleron GEXSCOPE, 10× Genomics, BD Rhapsody, and scCOOL-seq (**Table 1**). The advancements in these single-cell sequencing technologies are primarily evident in improved sensitivity, optimized data accuracy, and significantly increased sequencing throughput (26). From early technologies like STRT-seq and Smart-seq2 to later innovations such as 10× Genomics and GEXSCOPE, the continuous breakthroughs of these technologies have overcome traditional limitations, enabling single-cell sequencing to handle more complex and high-dimensional data analysis tasks, particularly in areas such as cellular heterogeneity analysis, low-abundance RNA detection, and multi-omics data integration. For example, Huang et al. (27) employed modified scCOOL-seq to uncover the transcriptome, DNA methylation, and chromatin accessibility landscapes of adult human testicular cells at single-cell resolution. Wu et al. (28) revealed the long-read transcriptional landscape of spermatogenesis in patients with OA and SCOS using PacBio long-read single-cell sequencing and short-read scRNA-seq.

Unlike the continuous innovation in sequencing technologies, the analytical methods for single-cell sequencing data have not undergone significant changes. The basic process for analyzing single-cell sequencing data begins with data preprocessing, including quality control and filtering of the raw sequencing data. Subsequently, through dimensionality reduction, clustering, and cell type identification, the goal of detecting differentially expressed genes between distinct cell clusters is achieved. This enables researchers to identify key signature genes and clarify cellular heterogeneity at the single-cell level. These analysis methods are employed in all the studies we reviewed. Depending on the research objectives, researchers may also conduct additional analyses, such as cell trajectory and pseudotime analysis, gene set enrichment analysis, cellular energy metabolism analysis, and cell-cell interactions analysis. The variation among these studies primarily depends on factors such as the pathological types of the NOA samples, sample size, and the specific cell clusters or biological processes being studied. For example, Zhao et al. (18) focused on three types of NOA pathology (iNOA, KS, and Y chromosome microdeletions) and emphasized the central role of Sertoli cells in spermatogenesis. In contrast, Mahyari et al.’s research mainly targeted KS and identified an epigenetic phenomenon in KS Sertoli cells (29). Wang et al. (30) investigated the autophagic homeostasis of germ cells during spermatogenesis, while Alfano et al. (31) focused on testicular somatic cell senescence and DNA damage. Despite these differences in research focus, the data analysis methods employed in these studies were generally consistent.

Despite the growing body of single-cell studies on NOA, several challenges persist in applying single-cell sequencing technology to this area. First, research on germ cells and rare cell populations (e.g., macrophages and mast cells) remains limited. Second, Sertoli cells, due to their large size and complex structure, are more susceptible to damage or loss during the preparation of single-cell suspensions, which can impact their representation in single-cell sequencing analyses (11). Third, physical methods for tissue isolation may induce a stress response in the cells, altering their molecular characteristics. Fourth, single-cell sequencing inherently lacks spatial information (9). Although this limitation can be mitigated by integrating with spatial transcriptomics, spatial single-cell sequencing typically involves specialized probes and imaging techniques, leading to higher research costs. Fifth, the heterogeneity of the pathological states in NOA testicular samples complicates the interpretation of the sequencing results. Despite these various limitations, it is expected that as single-cell sequencing technology continues to develop and be more broadly applied, an increasing number of studies based on testicular samples from patients with NOA will be published, incorporating more comprehensive analytical approaches.

### 4.1 Characteristics of NOA germ cells

Spermatogenesis is a complex, highly regulated process involving critical events such as the maintenance and differentiation of SSCs via mitosis, meiosis, and spermiogenesis (32). Each stage requires precise genetic and signaling regulation to ensure a continuous supply of spermatozoa, essential for male fertility (33). The identification of various germ cells through single-cell sequencing is essential for a comprehensive understanding of their development. However, traditional markers may not be suitable for single-cell sequencing. For instance, immunohistochemical staining revealed that spermatogonia and spermatocytes are DDX4 positive, while spermatids are DDX4 negative (34). Nevertheless, Wang et al. (7) found that among the DDX4-positive germ cells in the scRNA-seq dataset, nearly all types of human male germ cells were identified. In single-cell sequencing studies, these germ cells can be classified based on their developmental stages and corresponding markers: SSCs (GFRA1, UTF1), differentiating spermatogonia (KIT, MKI67), differentiated spermatogonia (STRA8), leptotene and zygotene spermatocytes (SPO11, SYCP1), pachytene and diplotene spermatocytes (OVOL1, OVOL2), meiotic I/II and secondary spermatocytes (ACR, C9orf116, SLC26A3), round spermatids (TEX29, SIRT1), and elongated spermatids (PMR1, PMR2, PMR3, SPEM1) (7, 9, 18). Notably, patients with NOA may experience maturational arrest (MA) of germ cells or even complete absence of germ cells, as seen in conditions such as SCOS.

Abnormalities in proliferation and apoptosis within germ cells are primary contributors to sperm developmental arrest or cell death. Genes associated with SSCs proliferation, such as FOXP4, MAGEB2, SPOCD1, CD164, LELP1, and TEX38, have been identified as exhibiting low expression in the germ cells of patients with NOA (25, 35–37). Chen et al. (25) performed scRNA-seq analysis of 3,696 single cells from 17 iNOA patients who had spermatogenic cells but no mature sperm. After integrating scRNA-seq data from 2,854 testicular cells of normal samples (external data), they found that in the germ cells of iNOA patients, genes associated with the cell cycle were upregulated, while genes related to energy metabolism and gametogenesis were downregulated. CD164, LELP1, and TEX38, with decreased expression in germ cells, may serve as potential pathogenic genes for iNOA. To investigate the role of CD164 in spermatogonia development, researchers utilized the GC-1 spg spermatogonia cell line derived from mice and successfully knocked down Cd164, resulting in a significant increase in apoptosis rates. Transcriptomic analysis revealed that Cd164 knockdown activated genes related to cell chemotaxis and migration while inhibiting differentiation genes. Moreover, the expression levels of several apoptosis-related genes, including Bim, Bak, Casp9, ASK1/JNK, and p53, were significantly elevated following Cd164 knockdown. These results suggest that CD164 downregulation triggers intrinsic apoptotic pathways and mGluR5-mediated Ca^2+^ signaling, potentially leading to spermatogonia apoptosis. Similar to CD164, the low expression of FOXP4, MAGEB2, and SPOCD1 also disrupts SSCs proliferation and apoptosis. Although this study includes the largest number of NOA cases among primary studies and has conducted functional validation of the identified key pathogenic genes using cell lines, further research using animal models is needed to more clearly define the impact of these pathogenic genes on spermatogenesis.

Dysregulation of specific germ cell autophagy-related gene clusters has also been implicated in NOA. Autophagy, a cellular self-degradation process, involves the transport of unwanted or damaged organelles and proteins to lysosomes for degradation and recycling. Disruption of autophagy genes can impair cellular function and stability and lead to reproductive disorders (30, 38). Wang et al. (30) provided a comprehensive view at single-cell resolution of how autophagy regulates spermatogenesis and confirmed the dysregulation of autophagy-related gene clusters in the testicular germ cells of patients with NOA. They sequenced 480 cells from one NOA patient and retained the cells that expressed more than 2,000 genes and contained 10,000 transcripts. A total of 432 cells from this NOA patient, along with 2,854 testicular cells from 9 spermatogenically normal male individuals reported in published studies, were used for subsequent analysis. In addition, scRNA-seq data from 6,490 normal human testicular cells and 53,510 normal mouse testicular cells were used to validate the candidate key genes. This study revealed a novel role of Cst3, which encodes a cysteine protease inhibitor, as an autophagy suppressor in maintaining autophagy homeostasis in mouse SSCs. A limitation of this study is that it only included scRNA-seq from a single NOA patient (432 cells). Although the researchers leveraged a substantial amount of published sequencing data (9,344 human testicular cells and 53,510 mouse testicular cells) to illustrate the autophagy regulatory network in normal spermatogenesis across humans and mice, the dysregulation of some clusters of autophagy-related genes in NOA requires validation in studies with larger sample sizes.

Additionally, alterations in the demethylation status of germ cell DNA have been linked to NOA. Huang et al. (27) performed single-cell multi-omics sequencing (scCOOL-seq: DNA methylation and chromatin accessibility, STRT-seq: transcriptome) on four normal donors and two patients with NOA (round spermatid stagnation and pachytene spermatocyte arrest) to assess DNA methylation abnormalities in testicular germ cells. A total of multi-omics sequencing data from 1,097 cells from normal donors and 206 cells from NOA patients were analyzed. An average of 9,323 genes, 2,344,974 WCG sites and 18,999,905 GCH sites were detected in each individual cell. They discovered that the decrease in DNA methylation upon meiosis initiation in human spermatogenesis might be passive DNA demethylation during DNA replication, and this round of DNA demethylation was correlated with male meiotic recombination rather than gene expression. Additionally, aberrant DNA hypermethylation could be detected in leptotene spermatocytes of certain nonobstructive azoospermia patients. DNMT1 and its chaperone UHRF1 are involved in maintaining DNA methylation (39). In this study, the increase of DNMT1 transcript in the leptotene cells was detected in 44.4% (4/9) of NOA cases, while the increase of UHRF1 transcript was detected in 22.2% (2/9) of NOA cases. Using the well-recognized UHRF1 loss-of-function and UHRF-DNMT1 overexpression mouse models, they demonstrated that the disruption of this round of DNA demethylation could significantly affect the formation of male meiotic DNA double-strand breaks and fertility.

### 4.2 The critical role of Sertoli cells in spermatogenesis

The Sertoli cells, first identified by Enrico Sertoli in 1865, is a crucial somatic cell in the testis, essential for both germ cells development and the maintenance of the testicular microenvironment (40). Located in the basement membrane of seminiferous tubules, Sertoli cells extend into the lumen and act as a scaffold, forming the blood-testis barrier that protects germ cells. They also provide metabolic and nutritional support, regulate spermatogenesis, and are integral to testicular functions, including the proliferation of SSCs and the release of mature spermatozoa (41). Moreover, Sertoli cells support leydig cells (LCs) development and the normal function of peritubular myoblasts, and help to maintain local immune tolerance in the testis (42, 43). Sertoli cells play a crucial role in the endocrine regulation within the testes, supporting spermatogenesis through their interactions with follicle-stimulating hormone (FSH) and testosterone (44). FSH stimulates Sertoli cells to secrete growth factors that promote the development of germ cells, while luteinizing hormone (LH) indirectly affects Sertoli cells function by increasing testosterone secretion from LCs. Testosterone further enhances Sertoli cells support for germ cells, maintaining an optimal microenvironment for spermatogenesis. Additionally, Sertoli cells regulate FSH secretion through the secretion of inhibin, ensuring endocrine balance to facilitate normal sperm production. In summary, the interactions between Sertoli cells, FSH, LH, and testosterone form a complex and highly coordinated system that is vital for maintaining male reproductive health and normal spermatogenesis. Dysfunction of Sertoli cells can impair their multiple supportive and endocrine regulatory roles. The specific distribution of Sertoli cells and their critical role in spermatogenesis highlight their importance in the study of male infertility (43).

Early studies indicated that Sertoli cells exist in two distinct states: immature and mature Sertoli cells, before and after puberty (45). With the advent of scRNA-seq, Guo et al. (17) discovered two distinct populations of Sertoli cells in the human testis before puberty. These populations represented different developmental physiological states, which later converged into a mature Sertoli cell population after puberty. Zhao et al. (18) profiled 88,723 individual testicular cells from 10 healthy subjects of various ages and 7 patients with one of the three most common types of NOA (iNOA, KS, and AZF_Del). Through somatic cell scRNA-seq data of 10 normal testes at different developmental stages, they proposed that Sertoli cells undergo three sequential developmental phases: (1) a proliferative active, stem-like stage; (2) a structural maturation and metabolic conversion stage; and (3) a final maturation stage. Their findings also revealed significant differences between Sertoli cells from normal adults and those from patients with three types of NOA, with Sertoli cells exhibiting the greatest disparities in patients with NOA compared to other somatic clusters. Further comparisons of Sertoli cells across the three NOA types showed that in congenital conditions like KS and Y-chromosome microdeletions, Sertoli cells are mature, but they exhibit abnormal immune responses. In contrast, in iNOA, Sertoli cell maturation is blocked (18). Moreover, Zhao et al. observed that Wnt/β-catenin signaling was activated in immature Sertoli cells and Sertoli cells from patients with NOA, but inhibited in mature Sertoli cells. This suggests that the Wnt pathway may play a crucial role in regulating Sertoli cell maturation. In other studies, Zhang et al. (46) investigated the cellular communication network between spermatogenic and somatic cells in the testis and found that Sertoli cells secrete the highest number of proteins, significantly contributing to the testicular microenvironment. Additionally, Chen et al. (47) found that Sertoli cells from NOA testes exhibit distinct transcriptomic features, but did not identify any evidence supporting that changes in Sertoli cells lead to the loss of germ cells. In this study, they obtained 2,632 testicular cells from one OA donor and 1,212 testicular cells from one NOA donor for scRNA-seq, and integrated these scRNA-seq data with results from public datasets (healthy: n=1; NOA: n=1; OA: n=2). It is worth noting that these data were obtained from three different sequencing platforms: Singleron GEXSCOPE, 10x Genomics, and Smart-Seq2. Additionally, the quality and quantity of Sertoli cells in the public datasets were low. As the largest cell type in the testis, Sertoli cells are susceptible to damage during sample processing and sequencing. Therefore, it is crucial to clearly specify the quantity, quality, and proportion of Sertoli cells when conducting analyses involving them.

SARS-CoV-2 may enter the male reproductive system via Sertoli cells, which could potentially affect male reproductive function. Angiotensin-converting enzyme 2 (ACE2), identified as a receptor for SARS-CoV-2, is expressed in human testes, specifically in Sertoli cells. Liu et al. (48) conducted scRNA-seq analysis on 853 male embryonic primordial germ cells (PGCs), 2,854 normal testicular cells, and 228 Sertoli cells from patients with NOA. Their results revealed that ACE2-positive cells are present in almost all testicular cell types, where Sertoli cells in normal testicular tissue exhibiting the highest expression levels and the greatest proportion of positive cells. Interestingly, ACE2 expression was significantly reduced in Sertoli cells from patients with NOA. Similarly, Shen et al. (49) analyzed published scRNA-seq data and found that ACE2 mRNA was expressed in both somatic and germ cells. In normal middle-aged men, approximately 9% of SSCs were ACE2-positive, while Sertoli cells exhibited significantly higher expression, with 90% of Sertoli cells being ACE2-positive. In contrast, they observed a higher ACE2-positive rate in the testes of infertile men compared with normal individuals. These findings suggest that ACE2 expression in Sertoli cells and germ cells may contribute to male reproductive disorders associated with SARS-CoV-2 infection.

### 4.3 Abnormal Leydig cells and inflammatory state of testicular tissue

LCs, located in testicular interstitial space, are responsible for testosterone production in response to LH (50). Together with Sertoli cells, LCs play a crucial role in maintaining testicular homeostasis. During the eighth week of embryonic development, LCs differentiate from mesenchymal cells located between the seminiferous tubules. The differentiation status of these mesenchymal cells can be characterized using single-cell sequencing techniques in combination with cellular morphology and marker gene expression. Progenitor Leydig cells (PLCs) express steroidogenic enzymes such as CYP11A1 and HSD3B2 but lack HSD17B6 expression. Immature Leydig cells (ILCs) are identified by the expression of DLK1, IGF1, and HSD11B1, while mature Leydig cells (MLCs) are characterized by high levels of LHCGR expression (31).

Mahyari et al. (29) observed a reduced proportion of MLCs in individuals with NOA (KS), with no clear pattern regarding PLCs and ILCs ratios, indicating a predominantly ILCs state. A similar trend was noted by Alfano et al. (30), who reported overexpression of DLK1 and downregulation of IGF2 in LCs from NOA (SCOS) individuals, further confirming the immature state of these cells. In this study, they obtained 3,880 cells from iNOA patients for scRNA-seq, and integrated these scRNA-Seq data with results from public datasets (6,490 cells from three healthy men). Furthermore, they also found decreased levels of circulating total testosterone in the iNOA men. The low levels of total testosterone may be associated with the immature status of LCs. On the other hand, LCs secrete factors that regulate the functions of myoid cells, Sertoli cells, and macrophages. The immature state of LCs may compromise their ability to modulate these cells. However, further experiments are needed to validate this hypothesis.

Furthermore, the immaturity of LCs may alter the local immune microenvironment, leading to elevated oxidative stress, triggering an inflammatory response. Notably, Alfano et al. (31) found an imbalance in basement membrane collagen deposition in SCOS and observed the presence of HMGB1 in the cytoplasm of Sertoli cells, LCs, and myoid cells. HMGB1, a prototypical damage-associated molecular pattern protein, is released in response to inflammatory stimuli, suggesting an ongoing inflammatory response. The chemokine CCL2, a key pro-inflammatory mediator, is upregulated in NOA, with LCs being the primary source of CCL2 in dysgenic testes (51). Inflammation frequently correlates with impaired spermatogenesis (52), and prolonged inflammatory states can predispose individuals to a higher risk of cancer. In the study by Alfano et al. (31), the biallelic and deregulated expression of the imprinted gene IGF2 in ILCs was identified, which aligns with the characteristics of a pre-tumorigenic environment.

## 5 Re-analysis of public/published NOA single-cell sequencing data

The use of publicly available data for secondary analysis indeed provides a cost-effective and efficient alternative, particularly given the high costs, technical complexity, and scarcity of NOA samples associated with generating single-cell sequencing data. There are several compelling reasons for leveraging public databases in secondary analysis. Firstly, they offer the opportunity to reanalyze original datasets using updated computational tools and algorithms, allowing researchers to explore new hypotheses or validate existing findings. For instance, by employing more precise cell clustering algorithms, researchers can delve deeper into the heterogeneity of cell types or utilize novel molecular pathway analysis techniques to better understand the molecular mechanisms underlying specific conditions. Furthermore, secondary analysis may provide an opportunity to explore biological questions that were not the primary focus of the primary studies, contributing new perspectives for understanding NOA. It is important to acknowledge and express gratitude for the invaluable contributions made by the researchers who have generated and shared these datasets. Their pioneering work has not only expanded our understanding of NOA but also provided a foundation for further exploration and innovation. Secondary analysis builds upon their efforts, helping to refine and extend the knowledge base, and we deeply appreciate the open sharing of these data.

However, while secondary analysis of publicly available datasets offers these advantages, there are also some limitations to consider. Secondary analysis may be susceptible to biases or technical discrepancies that stem from the original data collection process. For example, variations in sample size, sample selection, and experimental conditions across studies could affect the reproducibility and generalizability of findings. Therefore, it is essential for researchers to be mindful of these potential biases when performing secondary analysis.

**Figure 2:**
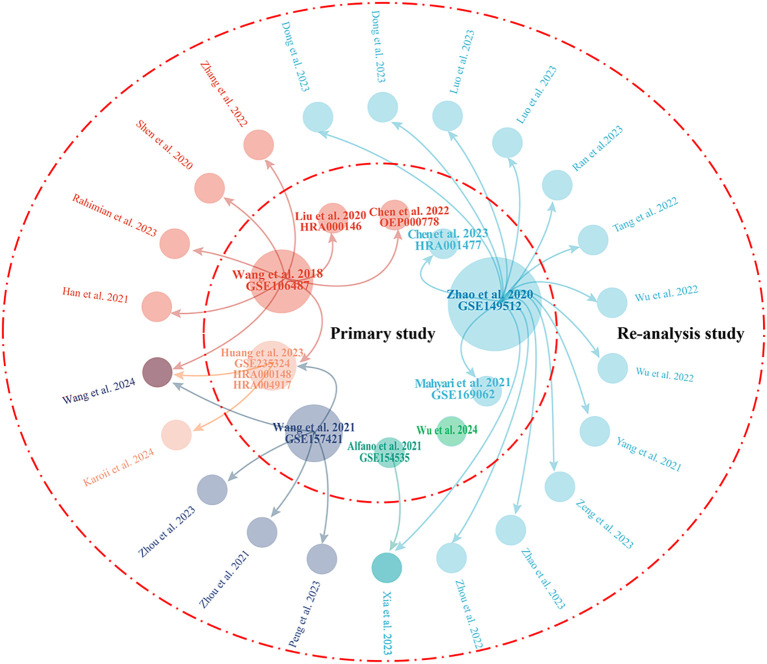
Visualization of the relationships between primary studies and re-analysis studies. The central circle represents the 10 primary studies, while the 22 outer circles denote re-analysis studies. The arrows indicate that the dataset has been re-analyzed in the studies to which they point. The size of the 10 circles representing the primary studies reflects the number of times they have been re-analyzed in other studies.

A total of 22 secondary studies were identified that used public/published NOA single-cell sequencing data for re-analysis (**Table 2**). Similar to the primary study, these studies were almost exclusively conducted by Chinese researchers. However, half of the re-analysis studies were published in 2023, followed by five in 2022, three in 2021, two in 2024, and one in 2020. In total, five scRNA-seq datasets were re-analyzed: GSE149512 (18), GSE106487 (7), GSE157421 (30), GSE235324 (27), and GSE154535 (31). Among these, the GSE149512 dataset, which includes data from ten healthy subjects of various ages and seven patients with NOA, emerged as the most frequently used dataset, re-analyzed in 15 studies within the current collection. The GSE106487 dataset was utilized in eight studies, GSE157421 in five studies, and GSE235324 and GSE154535 in two and one study, respectively. **Figure 2** illustrates the connections between primary and secondary studies.

**Table 2:** Studies with reanalysis of previously published scRNA-seq data of human NOA testicular samples.

Author	Year	Country	Data sources	Main findings from scRNA-seq data
Shen et al. (49)	2020	China	GSE106487	The mRNA expression of ACE2 was detected in both germ cells and somatic cells. SARS‐CoV‐2 may cause reproductive disorders through the pathway activated by ACE2.
Zhou et al. (53)	2021	China	GSE157421	A five-genes model for diagnosing NOA was constructed based on scRNA- seq of testicular tissue.
Yang et al. (67)	2021	China	GSE149512	ELAVL2, one of the RNA-binding proteins, was down-regulated in NOA. ELAVL2 promoted proliferation and inhibited apoptosis via activation ERK and AKT pathways in spermatogonia.
Han et al. (64)	2021	China	GSE106487	CHD5 and SPTBN2 may be potential biomarkers in the diagnosis and treatment of NOA.
Wu et al. (74)	2022	China	GSE149512	Defects in the microtubule cytoskeleton were noted in testes of NOA patients, possibly mediated by defective spatial expression and/or distribution of microtubule-associated proteins.
Tang et al. (58)	2022	China	GSE149512	Authors constructed transcriptional regulatory networks to explain the mutual regulatory relationship and the causal relationship between transcription factors. Specific TFs in NOA Leydig cells.: LHX9, KLF8, KLF4, ARID5B and RXRG. Specific TFs in NOA macrophages: POU2F2, SPIB, IRF5, CEBPA, ELK4 and KLF6.
Wu et al. (81)	2022	China	GSE149512	Local regulatory networks mediated by collagen and laminin chains may be disrupted in NOA.
Zhou et al. (37)	2022	China	GSE149512	SPOCD1 regulates the proliferation and apoptosis of human SSCs through adenylate kinase 4. Down-regulation of SPOCD1 may be associated with NOA.
Zhang et al. (46)	2022	China	GSE106487	Bisphenol A may lead to the dysregulation of secreted protein expression in Sertoli cells during spermatogenesis.
Zhou et al. (56)	2023	China	GSE157421	DDX20 and NCBP2 were all significantly associated with spermatogenesis.
Peng et al. (62)	2023	China	GSE157421	ETV2, TBX2, and ZNF689 were all significantly associated with spermatogenesis.
Ran et al. (63)	2023	China	GSE149512	C12orf54, TSSK6, OR2H1, FER1L5, C9orf153, and XKR3 were all located in spermatids.
Xia et al. (52)	2023	China	GSE149512 GSE154535	There are significant differences in cell proportions between iNOA samples and normal controls. In iNOA samples, the germ cells and Sertoli cells are markedly reduced. Additionally, heightened testicular inflammation associated with macrophages is evident in iNOA.
Zeng et al. (57)	2023	China	GSE149512	Wnt signaling was down-regulated in NOA spermatogonia. Three TFs (CTCF, AR, and ARNTL) may be involved in this dysfunctional Wnt signaling.
Dong et al. (51)	2023	China	GSE149512	CCL2 was identified as a functional testicular immune biomarker. Researchers drew a potential “myoid/Leydig cells-CCL2-ACKR1-endothelial cells-SELE-CD44-mast cells” network of somatic cell-cell communications in the testicular microenvironment, which might play roles in spermatogenic dysfunction.
Luo et al. (66)	2023	China	GSE149512	High expression of C9orf72 and CRTAP was observed in NOA macrophages. They may play critical roles in macrophage infiltration in NOA.
Dong et al. (68)	2023	China	GSE149512	CASP4 and GPX4, which are pyroptosis-related genes, might be functional in spermatogenic dysfunction.
Luo et al. (36)	2023	China	GSE149512	FOXP4 specifically marks a subset of spermatogonia with stem cell potential. FOXP4 in human SSCs lines suppressed cell proliferation and significantly activated apoptosis.
Zhao et al. (35)	2023	China	GSE149512	MAGEB2 is predominantly expressed in human SSCs. MAGEB2-mediated degradation of EGR1 regulates the proliferation and apoptosis of human SSCs lines.
Rahimian et al. (77)	2023	Iran	GSE106487	A novel missense variant in CDK5RAP2 was linked to NOA. The CDK5RAP2-MAPRE1 interactions may lead to germ cell centrosome defects.
Karoii et al. (65)	2024	Iran	GSE235324	Nine central genes associated with NOA spermatozoa were identified: RPL34, CYB5B, GOL6A6, LSM1, ARL4A, DHX57, STARD9, HSP90B1, and VPS36.
Wang et al. (61)	2024	China	GSE235324 GSE157421 GSE106487	Defective metabolic and transcription reprogramming were observed in NOA patient pachytene spermatocytes.

The primary research presents a comprehensive landscape of the molecular characteristics of NOA from the single-cell perspective, while the objectives of re-analysis studies are often more specific. For instance, Zhang et al. (46) analyzed scRNA-seq data from human juvenile testicular cells at different ages with the aim of confirming that Sertoli cells are a major driving force promoting germ cell maturity *in vivo*. Zhou et al. (53) analyzed scRNA-seq data from 432 NOA testicular cells with the aim of identifying marker genes for testicular cells to construct a diagnostic model of NOA. In addition to the analytical methods used in the primary studies, re-analysis studies often utilize some analytical techniques that are not specifically designed for scRNA-seq data, such as constructing protein-protein interaction (PPI) networks, weighted gene correlation network analysis (WCGNA), and least absolute shrinkage and selection operator (LASSO) Cox regression analysis. Furthermore, in re-analysis studies, research findings primarily rely on the bioinformatics analysis, which can process and reveal potential patterns and associations within large datasets. However, these findings often lack the necessary experimental or clinical validation, raising questions about the reliability of their conclusions. Therefore, although bioinformatics analysis can provide valuable preliminary insights, further experimental studies are required to support these findings and ensure their efficacy in clinical applications.

In conclusion, while secondary analysis of publicly available data presents certain challenges and limitations, they also offer unique opportunities for scientific discovery, particularly when resources are limited. By thoughtfully utilizing public databases, researchers can not only accelerate scientific progress but also provide valuable insights that can support further experimental design and clinical investigations. The following sections summarize the new insights gained from these re-analysis studies.

### 5.1 Abnormalities of transcriptional regulators of NOA

Transcriptional regulators (TRs), which include transcription factors (TFs) and chromatin regulators (CRs), are essential for the regulation of normal biological processes and are frequently implicated in various diseases (54, 55). Alterations in TR expression may contribute to the disruption of spermatogenesis. Zhou et al. (56) reported reduced expression of ETV2 and increased expression of TXB2 and ZNF689 in patients with NOA compared to controls, proposing these TRs as potential biomarkers for NOA. ETV2 is particularly associated with the development of endothelial cells and the formation of the blood-testis barrier, and its dysfunction may result in sperm damage (56). In Zhao et al.’s study (18), inhibition of the Wnt signaling pathway in Sertoli cells from patients with NOA has been shown to promote Sertoli cell maturation, thereby restoring their ability to support germ cell survival. Zeng et al. (56) re-analyzed Zhao et al.’s data (18), identifying three TFs (CTCF, AR, and ARNTL) with low expression in patients with NOA. They found that the abnormal expression of these TFs may lead to aberrant activation of the Wnt signaling pathway, ultimately impairing spermatogenesis. In another re-analysis of Zhao et al.’s data, Tang et al. (58) identified specific TRs expressed in testicular LCs and macrophages from patients with NOA, including LHX9, KLF8, KLF4, ARID5B, and RXRG in LCs, and POU2F2, SPIB, IRF5, CEBPA, ELK4, and KLF6 in macrophages.

Transcriptional reprogramming during meiosis in spermatocytes is essential for spermatogenesis, with TRs playing a pivotal driving role in this process (59, 60). Single-cell ATAC-seq (scATAC-seq) maps the chromatin accessibility landscape at the single-cell level, providing insights into cell-to-cell variability in gene regulation. Wang et al. (61) utilized scATAC-seq to uncover a critical transcriptional reprogramming event during mammalian spermatogenesis, occurring from the zygotene to pachytene transition (ZPT). This process is driven by the reproductive microenvironment and is regulated by TRs. They identified 282 TRs with changes in motif accessibility during ZPT. Further, the team re-analyzed published NOA single-cell data (GSE235321, GSE157421, and GSE106487) and found metabolic and transcriptional reprogramming defects in pachytene spermatocytes from patients with NOA. Notably, the key TRs regulated by Sertoli cell-derived signals in these cells were significantly down-regulated in the NOA samples, indicating a potential disruption of signaling communication between Sertoli cells and germ cells.

### 5.2 Abnormal RNA transcription of NOA

Re-analysis of publicly available or published single-cell sequencing data has revealed several potential pathogenic genes associated with NOA. These genes may serve as potential diagnostic markers for NOA. Down-regulation of genes such as DDX20, NCBP2, ODF2, CABYR, ELAVL2, C12orf54, TSSK6, OR2H1, FER1L5, C9orf153, XKR3, CHD5, SPTBN2, RPS4X, PSMD1, RPL36A, ARL4A, DHX57, STARD9, HSP90B1, VPS36, and GPX4 has been reported in patients with NOA (52, 53, 62–67). Conversely, up-regulation of genes including C9orf72, CRTAP, CASP4, RPL34, CYB5B, GOL6A6, and LSM1 has been observed (65, 66, 68).

Peng et al. (62) highlighted a significant decrease in the expression of DDX20 and NCBP2 in testicular tissues from patients with NOA. DDX20 is a member of the DEAD-box protein family, which is involved in RNA deconjugation, transcription, RNA modification, editing, localization, and maintenance of RNA stability. NCBP2 encodes a subunit of the nucleocapsid-binding protein complex, which regulates the processing and stability of mRNAs and miRNAs. Xia et al. (52) further found that CABYR and ODF2 exhibited significantly reduced expression in NOA, and their expression levels were negatively correlated with markers (e.g., CD86) of pro-inflammatory M1 macrophages. This suggests a potential link between inflammation and NOA pathogenesis. Similarly, Luo et al. (66) observed significant macrophage infiltration in NOA, with high expression levels of C9orf72 and CRTAP in these macrophages, supporting the hypothesis that inflammation contributes to NOA.

Yang et al. (67) identified the down-regulation of ELAVL2 in NOA tissues, which might reduce the stability and translational efficiency of the mRNAs associated with it. This reduction could impair the expression of proteins essential for regulating the proliferation and survival of SSCs. Ran et al. (63) introduced new marker genes for NOA, including C12orf54, TSSK6, OR2H1, FER1L5, C9orf153, and XKR3. C12orf54, TSSK6, and C9orf153 have been suggested to inhibit the MYC-related pathway, which is involved in cell cycle regulation and proliferation. Additionally, FER1L5 and C9orf153 may play a role in inhibiting the spermatogenesis pathway. Genes like RPS4X and RPL36A are implicated in ribosomal functions, while CHD5 may be associated with premature testicular aging, and SPTBN2 is involved in the regulation of the glutamate signaling pathway (53, 64). The upregulation of CASP4 suggests that cellular pyroptosis might be targeted as a therapeutic approach for spermatogenic dysfunction, making it a potential pharmacological target for treatment strategies (68).

### 5.3 The endocrine disruptor, bisphenol A, is an underlying cause of NOA

Bisphenol A (BPA) is a synthetic organic compound with estrogenic properties, commonly present in food and beverage containers, water bottles, and thermal paper receipts (69). BPA has the potential to disrupt endocrine function by interfering with hormone regulation and reproductive health. It can leach into food and water sources, leading to long-term exposure in humans (70). Zhang et al. (46) analyzed scRNA-seq data from 4,108 human juvenile testicular cells at different ages (7 years, 11 years, 13 years, and 14 years) (17) to confirm that Sertoli cells act as differentiation of germ cells promoting factors, which are largely mediated by receptor-ligand interactions. Subsequently, they found that BPA caused transcriptional dysregulation in Sertoli cells (genome-wide microarray data from Tabuchi et al.’ study) (71). These transcriptionally dysregulated genes partially overlap with the differentially expressed genes (NOA patients vs. donors with normal spermatogenesis) identified through scRNA-seq (DKK3, CCDC25, ACE2, and CCT3). Based on the aforementioned evidence, they reached the following conclusions: (1) BPA can change the expression of genes encoding secreted proteins in the testicular microenvironment; (2) BPA shares the common molecular pathway with NOA in the Sertoli cells through secreted proteins; (3) BPA had potential effects on spermatogenesis and the transcriptome of NOA. It should be noted that, in the single cell sequencing study by Guo et al. (17), 7,675 cells (contained > 500 expressed genes, and had < 25% reads mapped to mitochondrial genome) were retained for downstream analysis. In this study, a secondary analysis of Guo’s data was performed, and after quality control, only 4,108 cells remained (had < 20% reads mapped to mitochondrial genome). It would be best to provide the percentage of mitochondrial gene expression for all cells to explain why the number of cells passing quality control significantly decreased. Furthermore, the transcriptomic data used in the study were obtained from Sertoli cells exposed to 200 μM (45656 µg/L) BPA. Studies have shown that the BPA exposure in humans is significantly lower than 200 μM (72). Finally, changes at the transcriptomic level within cells does not equate to changes in protein levels, let alone reflect changes in the levels of secreted proteins outside the cells. Nevertheless, overlapping differentially expressed genes is not sufficient to indicate the existence of shared molecular pathways. This study integrated scRNA-seq data from NOA with transcriptomic data from Sertoli cells exposed to BPA to reveal the possibility that BPA may interfere with protein secretion in Sertoli cells, thereby participating in the pathogenesis of male infertility (NOA). This finding provides crucial scientific evidence for understanding the potential risks of environmental pollutants to reproductive health.

### 5.4 Defects in the microtubular cytoskeleton in patients with NOA

Microtubules (MT), composed of tubulin subunits, are dynamic structures integral to a variety of essential cellular processes (73). In Sertoli cells, MT provide structural support and function as tracks for intracellular cargo transport, as well as facilitating the transport of germ cells. In normal testes, the MT network forms a continuous track throughout the seminiferous epithelium, but in NOA testes, this track is markedly shortened and exhibits collapsed structures (74). Moreover, the localization of two MT-dependent motor proteins, dynein 1, which transports cargo toward the microtubule minus (-) end, and KIF15, which moves cargo toward the microtubule plus (+) end, becomes disordered in NOA testes. This disruption may result from the absence of intact MT-based tracks. Based on scRNA-seq data, Wu et al. (74) observed a slight down-regulation of dynein 1 in NOA testes and a significant alteration in the distribution of MAPRE1, a protein involved in stabilizing MT. The centrosome, as the microtubule-organizing center (MTOC), plays a critical role in microtubule formation, arrangement, and function, which are critical for the development, division, and chromosomal segregation of reproductive cells (75). CDK5RAP2, a centrosomal protein and an established biomarker for human azoospermia, regulates centrosome maturation by recruiting the γ-tubulin ring complex (γ-TuRC) to the centrosome. In collaboration with EB1/MAPRE1, CDK5RAP2 promotes MT polymerization at the centrosome, supporting MT bundling, growth, and dynamic behavior at the plus end (76). Rahimian et al. (77) identified a rare nonsynonymous single-nucleotide variant in CDK5RAP2 in NOA, which severely disrupts its interaction with MAPRE1, potentially leading to defects in germ cell centrosome. Dysfunctional centrosomal MT organization may contribute to spermatogenesis failure through mechanisms such as p53 signaling activation, DNA damage checkpoint response, and apoptosis (75, 77). Additionally, studies have indicated that environmental toxins induce damage to Sertoli cells and testes through alterations in the MT cytoskeleton, further highlighting the connection between MT abnormalities and NOA (78).

## 6 Conclusions and future perspectives

The primary objective of this review is to synthesize the latest insights derived from single-cell sequencing of human NOA testicular samples. This encompasses not only 10 primary studies, but also 22 re-analysis studies of previously published datasets. ScRNA-seq has contributed significantly to advancing our understanding of the pathogenic mechanisms of NOA, alterations in the testicular microenvironment, and the interactions between somatic and germ cells. Our summary highlights abnormalities in germ cells, Sertoli cells, LCs, TRs, transcription, and MT cytoskeleton within the testicular tissues of patients with NOA, shedding light on the potential pathogenesis of this condition. Furthermore, the increasing number of scRNA-seq studies on human NOA testes, alongside the continuous improvement of the technology, allows for the integration and re-analysis of published scRNA-seq data based on specific research objectives. Overall, this review serves as a comprehensive and detailed resource for researchers exploring NOA.

Despite its potential, there are some limitations associated with using single-cell sequencing in NOA research. These challenges include greater noise in the data, the absence of spatial structural information, and the limited number of studies focusing on rare cell populations (e.g., macrophages, T cells, mast cells, or even B cells) (79). In future applications, the two critical aspects that need to be emphasized for single-cell sequencing technologies are the pathological heterogeneity of NOA and the incorporation of multi-omics sequencing technologies. Patients with NOA exhibit significant heterogeneity in their testicular tissue, with marked differences in cell composition across various pathological types. For example, in patients with SCOS, the testicular tissue is composed solely of Sertoli cells, lacking germ cells; patients with spermatogenic maturation arrest may retain a small number of differentiated spermatogonia or spermatocytes, yet they are unable to complete meiosis; testicular tissue in patients with impaired spermatogenic function may contain germ cells at various developmental stages, yet their quantity and ratio are significantly reduced. Single-cell sequencing requires precise differentiation of various cell subpopulations (such as undifferentiated spermatogonia, Sertoli cells, and LCs) and interpretation of their functional states. However, the diversity in pathological types creates substantial discrepancies in cell type proportions between samples, complicating data integration and comparative analysis. Currently, single-cell research on NOA primarily focuses on transcriptomics, with limited application of multi-omics sequencing technologies. Relying solely on RNA expression is insufficient to comprehensively elucidate the effects of epigenetic regulation on spermatogenesis. For instance, meiotic arrest in NOA patients may be associated with abnormal DNA demethylation (27), yet these mechanisms are challenging to capture through RNA sequencing alone. The interplay between gene expression and epigenetic modifications during spermatogenesis necessitates the integration of multi-omics data. Certainly, the application of multi-omics sequencing faces significant challenges: (1) Technical complexity: The generation and analysis of multi-omics data are costly, and addressing the spatiotemporal synchronization between different omics data is necessary; (2) Sample scarcity: The limited number of testicular biopsy samples from NOA patients makes it difficult to support large-scale multi-omics cohort studies. In future research, it is essential to prioritize the construction of standardized databases, with the goal of establishing a multi-omics reference atlas encompassing various pathological types of NOA to facilitate data integration across studies. Furthermore, the introduction of multi-omics single-cell sequencing technologies, such as spatial epigenome–transcriptome co-profiling of mammalian tissues (80), is highly recommended, particularly suitable for studying cell heterogeneity and dynamic regulatory networks within the complex microenvironment of testicular tissue.

Single-cell studies in azoospermia, particularly NOA, have primarily focused on investigating fundamental scientific questions related to testicular tissue heterogeneity and mechanisms underlying germ cells developmental arrest. However, translating these findings into clinical applications presents a significant challenge. Building on existing research and clinical needs, future efforts should prioritize the following directions to drive translational progress: (1) Overcoming technical barriers. The clinical adoption of single-cell sequencing faces challenges including technical complexity, high costs, and difficulties in data analysis. Key strategies include developing clinically applicable multi-omics profiling technologies, optimizing sample processing techniques to address the challenges related to limited testicular biopsy sample volume and low cell viability, utilizing microfluidic platforms for enhanced cell capture, and establishing specialized bioinformatics pipelines that utilize AI-driven models to assist clinicians in identifying critical pathogenic genes or epigenetic markers. (2) Establishing precision subtyping and personalized therapeutic strategies: Develop clinical molecular subtyping based on single-cell sequencing to optimize surgical indications according to subtype results. Additionally, create a Nomogram scoring system based on single-cell data that integrates clinical parameters such as hormone levels and testicular volume to predict patient responses to hormonal therapy or surgery. Targeted therapeutic approaches may also be devised, such as using Wnt pathway inhibitors to address maturation defects in Sertoli cells associated with iNOA (18). The ultimate goal is to incorporate single-cell research findings into existing diagnostic and therapeutic frameworks, facilitating updates to clinical pathways and guidelines.

In summary, the application of single-cell sequencing technology in NOA has revolutionized our understanding of this condition, providing unprecedented insight into its pathological complexity. To translate these discoveries into clinical practice, critical steps include overcoming technical limitations, integrating multi-omics data, and validating findings through rigorous clinical trials. With breakthroughs in spatial multi-omics, AI-driven analytical tools, and stem cell technologies, single-cell research is poised to transition from bench to bedside. This evolution will empower clinicians to implement comprehensive treatment solutions for patients with NOA, ranging from molecular subtyping to personalized therapeutic interventions.
